# Feasibility and application of polygenic score analysis to the morphology of human-induced pluripotent stem cells

**DOI:** 10.1007/s00438-022-01905-2

**Published:** 2022-05-28

**Authors:** Jonathan R. I. Coleman

**Affiliations:** 1 Social, Genetic and Developmental Psychiatry Centre, Institute of Psychiatry, Psychology and Neuroscience-PO80, King’s College London, DeCrespigny Park, Denmark Hill, London, SE5 8AF UK; 2 National Institute for Health and Care Research Maudsley Biomedical Research Centre, South London and Maudsley NHS Foundation Trust, London, UK

**Keywords:** Human-induced pluripotent stem cells, Polygenic, Complex traits, Morphology

## Abstract

Genome-wide association studies have identified thousands of significant associations between genetic variants and complex traits. Inferring biological insights from these associations has been challenging. One approach attempted has been to examine the effects of individual variants in cellular models. Here, I demonstrate the feasibility of examining the aggregate effect of many variants on cellular phenotypes. I examine the effects of polygenic scores for cross-psychiatric disorder risk, schizophrenia, body mass index and height on cellular morphology, using 1.5 million induced pluripotent stem cells (iPSC) from 60 European-ancestry donors from the Human iPSC Initiative dataset. I show that measuring multiple cells per donor provides sufficient power for polygenic score analyses, and that cross-psychiatric disorder risk is associated with cell area (*p* = 0.004). Combined with emerging methods of high-throughput iPSC phenotyping, cellular polygenic scoring is a promising method for understanding potential biological effects of the polygenic component of complex traits.

**Supplementary Information:**

The online version contains supplementary material available at 10.1007/s00438-022-01905-2.

## Introduction

Genome-wide association studies (GWAS) have successfully identified statistical associations with common genetic variants, with over 325,000 variant–trait associations listed in the NHGRI-EBI GWAS catalog as of January 2022 (Buniello et al. 2019). In comparison with earlier methods such as genome-wide linkage analysis, GWAS have been effective in implicating variants associated with complex traits, and in highlighting their polygenic component (Cannon and Keller 2006; Visscher et al. 2017).

Gaining insights into the biology of complex traits has been a main motivator for GWAS. However, the promise of new biology has been slow to emerge (Visscher et al. 2017). Some of these limitations are inherent to GWAS, such as the difficulty of determining which of the numerous correlated variants associated with a trait at a given locus contribute to causality. Others reflect the limits of model systems. Animal models are useful for understanding the function of conserved genes within a living organism. In contrast, they are less useful when seeking to translate the effects of variants, because causal variants are poorly conserved across species (Flint and Mackay 2009). Studying certain phenotypes introduces further limitations. Perhaps the clearest example of this is the study of behaviour, where the brain is the focus of biological interest (Sullivan and Geschwind 2019). There are obvious ethical and logistical impediments to accessing living human brain tissue, which largely prevent direct functional experiments that would provide vital context for understanding and validating behavioural GWAS results.

Efforts to address these limitations of biological context are emerging, such as through the work of the PsychENCODE consortium in developing large datasets of biological annotations from post-mortem human brains (PsychENCODE Consortium 2018). A further promising area is the increasing diversity in neuron types derived from human-induced pluripotent stem cells (iPSCs; reviewed in (Fernando et al. 2020)). Mature human cells can be reprogrammed to a pluripotent state (iPSCs) by controlled exposure to specific transcription factors (Ebben et al. 2011). Self-renewing iPSCs can be maintained under standardised conditions (Efthymiou et al. 2014). Further treatment can direct the subsequent differentiation of the iPSCs down specific developmental pathways. For example, highly pure populations of all major neuron types in the human brain can now be derived from iPSCs (Fernando et al. 2020). Human iPSCs are a valuable model system when directly studying relevant tissue is challenging, as they enable the assessment of living human cell types. Derived cells only approximate the cell types they model; for example, derived neurons are developmentally immature, resembling foetal developmental stages (Fernando et al. 2020). In vivo cells reflect the developmental history of the organism (including the effects of intrinsic and extrinsic environments), a history that is not transferred to iPSCs when they are generated. However, for the purposes of modelling diseases, these approximations can be useful simplifications (Fernando et al. 2020).

As such, iPSCs can be a valuable model system in studying complex behavioural traits. To date, most genetic research using iPSCs in behavioural phenotypes has focussed on the effects of single variants of moderate effect, primarily in schizophrenia and autism spectrum disorder, where such effects have been shown to have an important contribution (Sullivan and Geschwind 2019; Vadodaria et al. 2020). However, most of the genetic contribution to behavioural traits is polygenic. It would be valuable to also be able to use iPSCs to examine polygenic components. Polygenic effects can be assessed through polygenic scoring, wherein multiple genetic variants are used to create a single score in an individual as a weighted sum of the alleles they carry (Lande and Thompson 1990; International Schizophrenia Consortium et al. 2009). Typically, the weights for polygenic scoring are derived from a base GWAS of a trait of interest, providing a means by which to assess the shared genetic variance between the trait of interest from the base GWAS and a phenotype in a target genotyped cohort (Choi et al. 2020). Polygenic scores have been widely applied at the population level in humans and in livestock (Wray et al. 2019), but have not yet been widely applied to examine genetic effects on cellular phenotypes.

In this exploratory paper, I aim to demonstrate the feasibility of polygenic scoring in iPSCs. I first use power calculations to assess the number of cell donors required for ≥ 80% power to detect plausible levels of genetic covariance between a GWAS and a cellular phenotype. I then test the association of polygenic scores from GWAS of complex traits with iPSC cell morphology. These GWAS are the most powerful available studies of a single psychiatric disorder [schizophrenia; (Pardiñas et al. 2018)], of shared genetic effects on psychiatric disorders (Cross-Disorder Group of the Psychiatric Genomics Consortium et al. 2019), and of other complex traits, one partially behavioural (body mass index; (Yengo et al. 2018)) and one without a behavioural aetiology (height; (Yengo et al. 2018)).

Donor number is among the principal limitations of polygenic analyses in iPSCs and their derivatives. Polygenic scores in complex traits typically capture only a small amount of variance, and so usually require hundreds of participants for sufficient power (Choi et al. 2020). I address this in two ways. I use data from the Human Pluripotent Stem Cell Initiative (HipSci), an openly-accessible, large collection of iPSCs generated with a standardised pipeline, with extensive phenotypic and genetic data (Kilpinen et al. 2017). I also use mixed linear models to analyse numerous cells from each donor. Although these technical replicates do not provide as much power as new donors, they provide some increase because they limit the impact of measurement error (Blainey et al. 2014).

I focus my analyses on the morphology of iPSC cells, due to the availability of sufficient data on these cells compared to their neuronal derivatives. It also is feasible that the polygenic components studied may affect the morphology of iPSCs directly. Previous analyses of the HipSci dataset have shown that cell-to-cell variation in morphology is attributable both to cell-extrinsic effects (such as the culturing environment) and to intrinsic effects, which may include the action of genetic variation between different cell lines (Vigilante et al. 2019). In this analysis, I aim to examine whether these intrinsic effects partly reflect specific polygenic components associated with variation in organismal-level biological processes, including body shape and behaviour. Genes involved in neuronal morphology have been implicated in numerous GWAS of psychiatric disorders (Wray et al. 2018; Cross-Disorder Group of the Psychiatric Genomics Consortium et al. 2019; Trubetskoy et al. 2022; Mullins et al. 2021), as have pathways involved in general cell morphology, such as cell–cell adhesion (Levey et al. 2021). While strongest in neurons, these effects may also be apparent in general cellular morphology, and particularly in iPSCs, whose cellular fate is undetermined. As such, investigating the role of complex trait polygenic scores in cell morphology is both a pragmatic demonstration of the broader promise of polygenic scoring in cells, and a question with biological relevance of its own.

## Materials and methods

### Human-induced pluripotent stem cell initiative (HipSci)

This paper consists of secondary analyses of existing data from HipSci. Full descriptions of the generation of these data are provided elsewhere (Leha et al. 2016; Kilpinen et al. 2017; Vigilante et al. 2019). I used data on genome-wide genotyping and cellular microscopy phenotyping from the HipSci project. HipSci comprises several disease cohorts, as well as a cohort of unaffected donors comprised of consented research volunteers from the National Institute of Health and Care Research Cambridge Bioresource. Data from unaffected donors were used in this study. Broader phenotypic information (such as height, BMI or mental health phenotyping, which would be relevant to the polygenic score analyses) was not available on these donors. Following quality control (described below), data from 103 iPSC cell lines from 60 donors were included in the final analysis (Supplementary Table 1, Supplementary Figure 1). All but two cell lines were included in a previous publication (Vigilante et al. 2019).

### Cellular phenotyping

Cellular phenotyping data were available for all analysed cell lines, and is described in detail elsewhere (Leha et al. 2016; Vigilante et al. 2019), and in brief in the Supplementary Methods. Three morphological phenotypes were determined from cell image data gathered by an Operetta (Perkin Elmer) high content device: cell area, roundness, and width-to-length ratio (Vigilante et al. 2019). Cell width-to-length ratio was defined as the length (the longest line that could be drawn within the cell) divided by the width (the longest line perpendicular to the length that could be drawn within the cell). Cell roundness was defined from Harmony image analysis using the equation below, where area and perimeter are defined analytically from cell images (Leha et al. 2016; Vigilante et al. 2019):

$$\mathrm{Roundness }= 3.544 \times \sqrt{\frac{\mathrm{area} - (\mathrm{perimeter}/2)}{\mathrm{perimeter} - 0.1}}.$$

### Genome-wide genotype data

Genome-wide genotype data were available for all analysed cell lines from the Illumina HumanCoreExome-12 v1 BeadChip, imputed to Haplotype Reference Consortium panel release 1.1. Full details of genotype quality control and imputation are provided in the Supplementary Materials. In summary, I retained a single cell line for each of the 60 donors for polygenic risk scoring. All donors were unrelated (pi-hat < 0.125, where pi-hat is a measure of genetic relatedness, and 0.125 is the relatedness between first cousins), well genotyped (call rate ≥ 0.99), and from European ancestries. I retained variants with MAF ≥ 0.05 that were directly genotyped or imputed with INFO ≥ 0.9.

### Polygenic scoring

I generated polygenic scores using PRSice v2.3.1e (Choi and O’Reilly 2019). I obtained summary statistics for height and BMI GWAS (Yengo et al. 2018) from the GIANT consortium, and for schizophrenia (Pardiñas et al. 2018) and cross-psychiatric disorder GWAS (Cross-Disorder Group of the Psychiatric Genomics Consortium et al. 2019) from the Psychiatric Genomics Consortium. The cross-psychiatric disorder GWAS captures shared genetic effects from GWAS of schizophrenia, bipolar disorder, major depression, autism spectrum disorder, attention-deficit hyperactivity disorder, obsessive–compulsive disorder, anorexia nervosa, and Tourette’s syndrome. The method used in the cross-psychiatric disorder GWAS results in equal contributions from each disorder, despite differences in power between the GWAS included.

For each base GWAS, I calculated polygenic scores limiting to autosomal variants in common between each set of summary statistics, the HipSci data, and the non-Finnish European participants in 1000 Genomes (1000 Genomes Project Consortium et al. 2015). I removed variants in linkage disequilibrium (*r*^2^ > 0.1) with a variant with a lower p-value within 250 kilobases. The HipSci cohort is small for polygenic score analysis, so I estimated the linkage disequilibrium structure from the 404 non-Finnish European participants in 1000 Genomes (1000 Genomes Project Consortium et al. 2015; Choi et al. 2020). Exploratory polygenic scoring is typically performed using multiple scores made up of variants with a p value in the base GWAS smaller than a given threshold. For example, the PRSice software generates eight scores by default, at *p* < 0.001, 0.05, 0.1, 0.2, 0.3, 0.4, 0.5, and 1. For each base GWAS, I generated a polygenic score at the threshold capturing the greatest proportion of variance in leave-one-sample-out polygenic scoring from the initial publications (see Supplementary Methods for a description of the calculation of the proportion of variance). For schizophrenia, the threshold at which the most variance was captured was *p* < 0.05 (which captured 5–6% of variance in the original publication; Pardiñas et al. 2018). For height and BMI, the threshold at which the most variance was captured was *p* < 0.001 for both phenotypes (capturing 24% and 10% of variance respectively in the original publication; Yengo et al. 2018). I also generated a polygenic score at *p* < 1 to capture the effects of all variants (at the expense of including noise from variants not truly associated with the trait). Leave-one-sample-out polygenic scoring was not reported in the original cross-psychiatric disorder publication. Accordingly, I generated three polygenic scores for the cross-psychiatric disorder data: (1) including data from 23andME, limited to 10,000 variants in linkage equilibrium with *p* < 0.001; (2) excluding 23andME and limiting to *p* < 0.001 for comparison; and (3) excluding 23andME and including all variants (*p* < 1). Polygenic scores are referenced in the format Trait_Threshold_, for example Schizophrenia_0.05_ (Table 1).

### Power analyses

To assess the current power for polygenic score analyses in iPSCs, I performed a series of power analyses in R version 3.6, using the AVENGEME package (Supplementary Methods) (Core Team and Others 2013; Palla and Dudbridge 2015). I performed power analyses for each of the full polygenic scores (that is, those including all variants, *p* < 1) used in this paper, assessing the power of analyses assuming iPSCs from 60 donors. These analyses do not take account of the multiple phenotypic measurements made for each donor, and so underestimate power. Taking into account the multiple phenotypic measurements results in an effective N of 850–2435 (Supplementary Methods; Supplementary Table 2). I ran additional power analyses using these two values.

I defined parameters for power analysis from the GWAS from which each polygenic score originated (Supplementary Methods; Supplementary Table 3). I estimated the power of polygenic score analysis for a hypothetical cellular phenotype, varying the covariance between the polygenic score trait and the phenotype. Covariance can be described as a function of the common genetic contribution to the polygenic score trait (vg1, which is fixed), the common genetic contribution to the phenotype (vg2, which I varied), and the genetic correlation between the polygenic score trait and the phenotype (which I varied):

$$Covariance =Correlation * \sqrt{vg1 \times vg2}.$$

### Statistical analysis

I ran all analysis in R version 3.6 (Core Team and Others 2013). I restricted the data for analyses to 103 cell lines (from 60 donors) for which cell morphological data and polygenic scores were available (Supplementary Table 1, Supplementary Figure 1). Cell morphological phenotype data were available for 1,543,624 individual cells in 2484 plate wells. I also considered the effect of the fibronectin concentration (coded as an ordinal variable of 1, 5, 25) of the wells on which cells were plated as a variable of interest, as this has been shown to contribute importantly to cell morphology (Vigilante et al. 2019). I controlled for continuous variables of four genomic principal components (to control for population stratification), and the number of cells in each clump, as well as for factors assessing the origin cell for the iPSC (fibroblasts or peripheral blood mononuclear cells), the method of iPSC reprogramming (episomal DNA or Sendai vector), and whether or not the origin cell was maintained on a feeder. All of these may affect cell morphology.

**Fig. 1:**
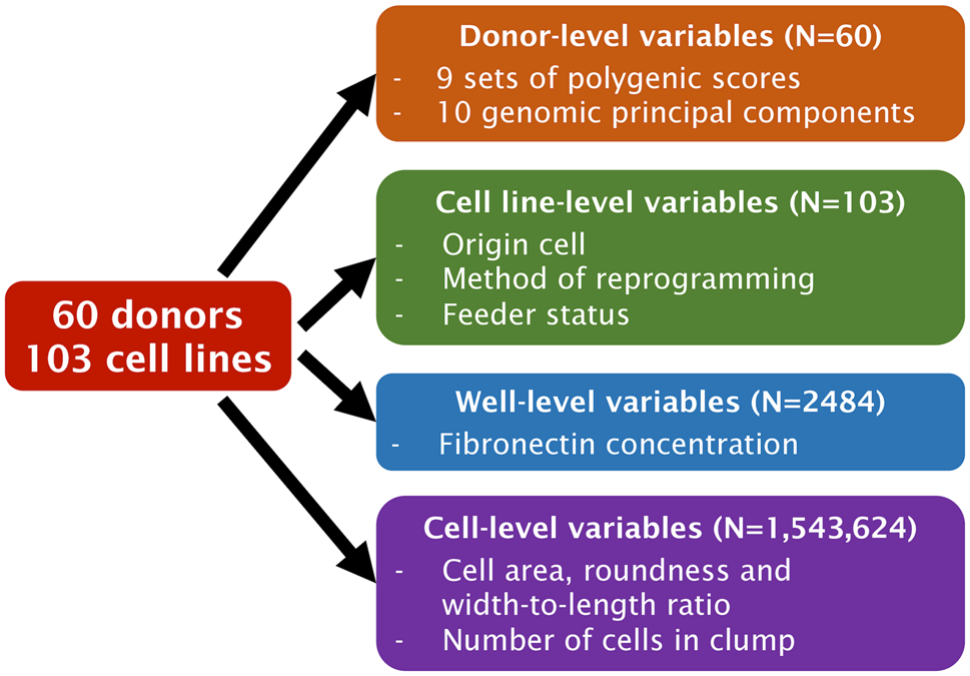
Source of variables for analysis, and sample size at each level of analysis

Data were drawn from different, nested levels of analysis (Fig. 1). Accordingly, I calculated associations between polygenic scores and cell shape phenotypes using mixed linear models from the *lmerTest* package (Kuznetsova et al. 2017). I included all variables described above as fixed effects, alongside a random intercept term of plate well nested within donor. As each well contains only cells from a single cell line, the random effect of well also captures variance between cell lines from the same donor. This allows for all observations to be included while controlling for pseudoreplication resulting from measuring multiple cells from each donor.

I performed analyses with and without assessing the interaction between the variables of interest (polygenic score and fibronectin concentration). In analyses including this interaction, I included additional interaction terms between each of the variables of interest and all covariates (Yzerbyt et al. 2004; Keller 2014). I fit all mixed linear models using REML and calculated the significance of coefficients using Satterthwaite's approximation for degrees of freedom. I visualised interaction models using the R packages *ggplot2* and *interactions* (Wickham 2016; Long 2019).

I standardised all continuous variables within-cohort prior to analysis. I included all other variables as binary factors. As such, the effect size (B) for polygenic scores can be interpreted as the change in standard deviations in the phenotype for one standard deviation change in the polygenic score. The effect size for fibronectin concentration can be interpreted as the change in standard deviations in the phenotype when comparing the tested conditions (either 5 or 25 μg/mL concentration) to the baseline condition (1 μg/mL).

### Sensitivity analyses

I performed several sensitivity analyses to assess the robustness of the model. First, I assessed the significance of the polygenic score effects as a likelihood ratio test comparing the mixed linear models (fit with maximum likelihood) with and without the polygenic score. For the models including polygenic score-by-fibronectin concentration interaction terms, I compared models with and without these interaction terms (but including the main effects of polygenic score and fibronectin concentration). Second, to assess the contribution of individual donors to the observed associations, I ran leave-one-donor-out models for all models without interaction terms. Finally, I assessed the importance of the analytical choices made in coding and including certain covariates on the statistically significant finding reported. Specifically, I varied the number of genomic principal components in the model (comparing models with 2 principal components and 10 principal components), and I separately recoded the number of cells in each clump as an ordinal variable [1 cell (single, reference), 2 or 3 cells (multiple, no cells surrounded by other cells), 4 or more cells (multiple, cells surrounded by other cells)].

### Multiple testing correction

In total, I assessed the association of nine non-independent polygenic scores with three non-independent phenotypes. To assess the effective number of tests incurred, I performed principal component analysis on the pairwise correlation matrices of the polygenic scores and the phenotypes separately. I defined the effective number of tests incurred as the number of principal components required to account for 99.5% of the variance in the correlation matrices. This resulted in 12 effective tests in total (6 effectively independent polygenic scores, 2 effectively independent phenotypes; Supplementary Table 4). As such, statistical significance was set at *p* < 4.16 × 10^–3^ (i.e., 0.05/12).

## Results

### Power analyses

**Fig. 2:**
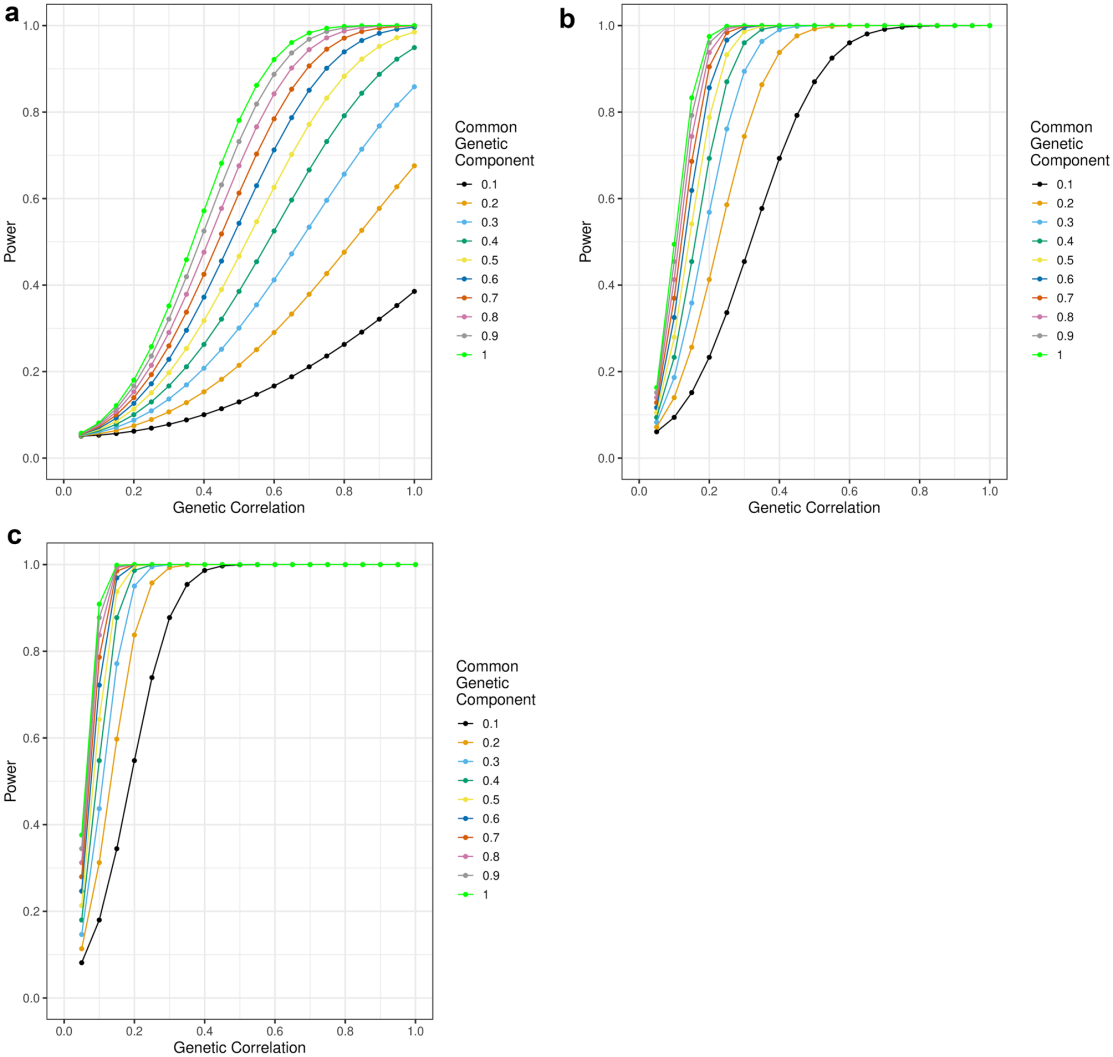
Power (y axis) to detect a genetic relationship between Cross-psychiatric_1_ and a cellular phenotype with a common genetic component of varying size (coloured lines) at different values of genetic correlation (*x*-axis), for differing values of *N*: **a** 60 [not accounting for multiple measurements], **b** 850 [lower estimate of effective *N*], **c** 2435 [higher estimate of effective *N*]

Power analyses examined the power of different numbers of donors to detect significant associations between a hypothetical cellular phenotype and the polygenic scores used in this analysis. Polygenic scores are referenced in the format Trait_Threshold_, for example Schizophrenia_0.05_ (Table 1). Results for the cross-psychiatric polygenic score using all variants with *p* < 1 (Cross-psychiatric_1_) are described here (Fig. 2, Supplementary Table 3). Results for other polygenic scores are described in the Supplementary Results (Supplementary Table 3, Supplementary Figures 2–4).

At the current donor number (*n* = 60), analyses only have ≥ 80% power when the genetic covariance between Cross-psychiatric_1_ and the cellular phenotype was ≥ 0.26. This could correspond to a genetic correlation ≥ 0.7 when the cellular phenotype has a SNP-based heritability ≥ 0.55 (Fig. 2a). Note that these values of SNP-based heritability and genetic correlation (as well as others given below) are example values that would correspond to this value of genetic covariance—alternative examples can be seen in Supplementary Table 3. A genetic covariance ≥ 0.26 between the cellular phenotypes and organism-level complex traits being examined herein is unrealistically high, suggesting that analyses are underpowered when setting n as the donor number.

However, setting n as the donor number does not take into account the potential power gain from measuring multiple cells from the same donor. Taking this into account yields estimates of effective *n* ranging 850–2435. At these sample sizes, analyses have ≥ 80% power when the genetic covariance between Cross-psychiatric_1_ and the cellular phenotype was ≥ 0.04–0.07. This could correspond to a genetic correlation ≥ 0.15–0.2 when the cellular phenotype has a SNP-based heritability ≥ 0.55, or a genetic correlation ≥ 0.3–0.5 when the SNP-based heritability is ≥ 0.1 (Fig. 2b, 2c).

### Analyses without interactions

I assessed the association of each polygenic score with morphological variability between cells, assessing cell area, cell roundness, and cell width-to-length ratio. Across 27 analyses, one polygenic score was significantly associated (*p* < 4.16 × 10^–3^) with a cell morphological phenotype (Cross-psychiatric_1_ associated with cell area, *B* = 0.085, *p* = 3.59 × 10^–3^; Table 1; full models in Supplementary Table 5). This association persisted in all sensitivity analyses (Supplementary Results; Supplementary Table 5). As expected, fibronectin concentration had a strong effect on all cell morphological phenotypes (absolute B 0.124–0.744, *p* < 10^–10^; Supplementary Table 5). *P* values obtained from the likelihood ratio test were consistent with those using Satterthwaite's approximation (Supplementary Table 5).

**Table 1:** Main effects of polygenic scores on cellular phenotypes

Polygenic score	Area beta	Area SE	Area P	Roundness beta	Roundness SE	Roundness P	Width:length beta	Width:length SE	Width:length P
Cross-psychiatric_0.001_	0.0068	0.0281	0.810	−0.0261	0.0345	0.454	−0.0210	0.0212	0.327
Cross-psychiatric_1_	**0.0845**	**0.0277**	**0.00359**	−0.1026	0.0345	0.00441	−0.0425	0.0223	0.0617
Cross-psychiatric_23andMe_	0.0100	0.0297	0.737	−0.0305	0.0365	0.407	−0.0218	0.0224	0.335
Schizophrenia_0.05_	−0.0131	0.0295	0.660	0.0171	0.0364	0.640	−0.0128	0.0224	0.572
Schizophrenia_1_	−0.0008	0.0263	0.975	0.0099	0.0323	0.761	−0.0140	0.0199	0.486
BMI_0.001_	−0.0186	0.0330	0.577	0.0033	0.0408	0.935	−0.00198	0.0252	0.938
BMI_1_	−0.0222	0.0299	0.461	−0.0331	0.0367	0.372	−0.0377	0.0223	0.0972
Height_0.001_	0.0644	0.0301	0.0371	−0.0592	0.0379	0.124	−0.0323	0.0236	0.176
Height_1_	0.0467	0.0293	0.116	−0.0585	0.0361	0.111	−0.0185	0.0227	0.419

Polygenic scores are referenced as Trait_Threshold_. Betas are on the scale of standard deviation changes in phenotype for one standard deviation change in polygenic score. Significant associations shown in bold (*p* < 0.00416). All values drawn from multiple linear regressions, full models in Supplementary Table 5.

### Analyses with interactions between polygenic scores and fibronectin concentration

**Fig. 3:**
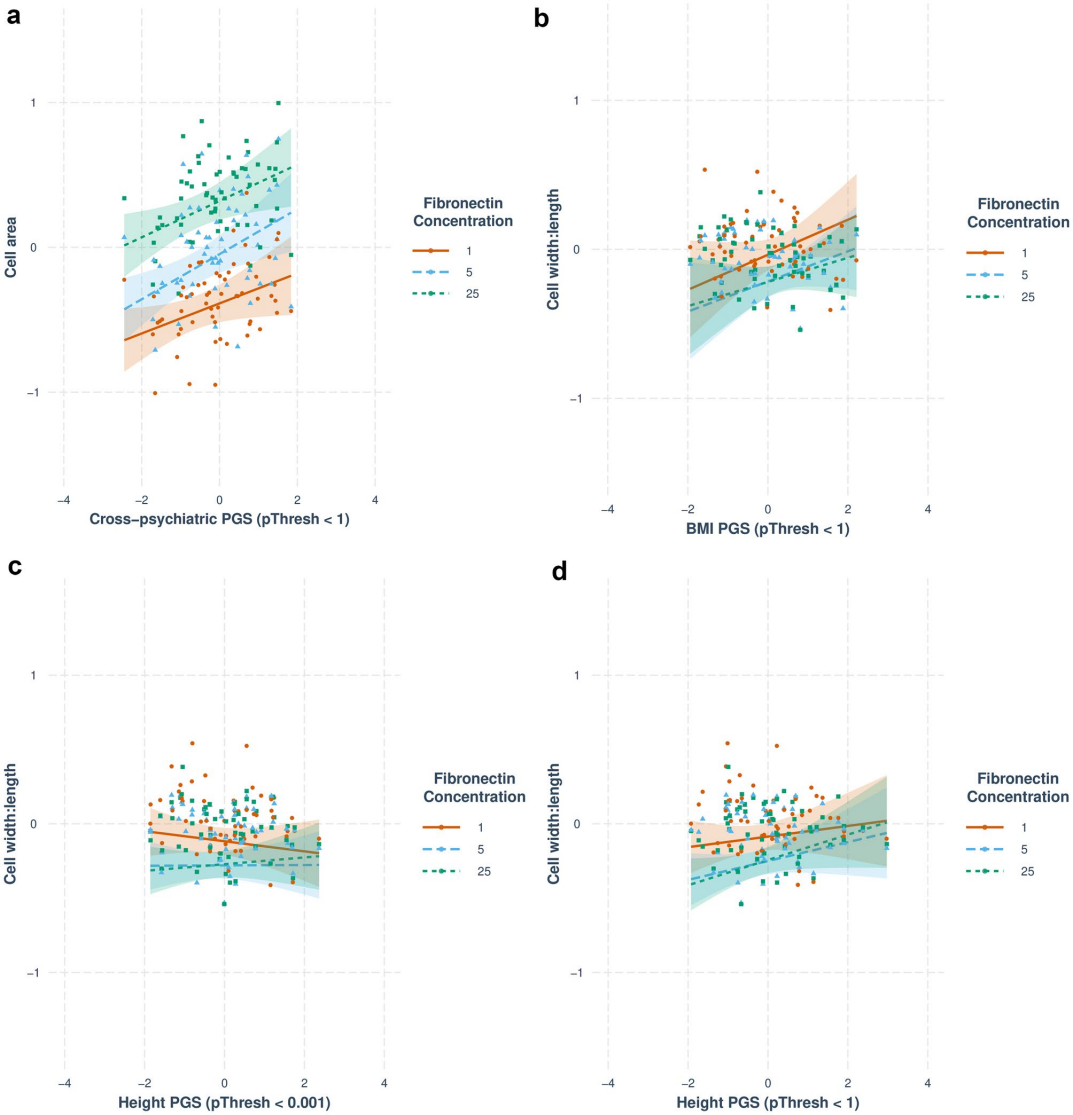
Significant interactions between polygenic scores and fibronectin concentrations. Lines reflect the relationship between each polygenic score and the phenotype from the relevant model. Points reflect the fitted value of the phenotype from the relevant model, averaged for each value of the polygenic score (that is, for all cells from a given donor). **a** Cross-psychiatric_1_ and fibronectin concentration of 5 μg/mL on cell area, **b** BMI_1_ and fibronectin concentration of 25 μg/mL on cell width-to-length ratio, **c** height_0.01_ and fibronectin concentration of 25 μg/mL on cell width-to-length ratio, **d** height_1_ and fibronectin concentration of 25 μg/mL on cell width-to-length ratio. *PGS* polygenic score, *BMI* body mass index

I then assessed how each polygenic score altered the known effect of fibronectin concentration on cellular morphology. Four significant interaction terms were observed between fibronectin concentration and polygenic scores (Fig. 3; Table 2; Supplementary Table 6). Of most interest is the interaction between Cross-psychiatric_1_ and fibronectin concentration of 5 μg/mL on cell area (*B* = 0.052, *p* = 5.61 × 10^–4^; Fig. 3a). This suggests the effect of Cross-psychiatric_1_ should be interpreted in the context of differing fibronectin concentrations. Specifically, the effect of plating cells on 5 μg/mL fibronectin (compared to a suboptimal concentration of 1 μg/mL) on increased cell area is greater in cells with higher Cross-psychiatric_1_ polygenic scores. Further significant interactions were seen in the analysis of cell width-to-length ratio, between a fibronectin concentration of 25 μg/mL and BMI_1_ (*B* = −0.037, *p* = 2.70 × 10^–3^; Fig. 3b), Height_0.01_ (*B* = 0.057, *p* = 5.65 × 10^–6^; Fig. 3c), and Height_1_ (*B* = 0.050, *p* = 3.70 × 10^–5^; Fig. 3d) respectively. In the absence of a main effect of these polygenic scores on cell width-to-length ratio, and the weaker effect of fibronectin on cell width-to-length ratio compared to cell area, these results are harder to interpret than the interaction described above. *P* values obtained from the likelihood ratio test were consistent with those using Satterthwaite's approximation (Supplementary Table 6).

**Table 2:** Effects of polygenic score-by-fibronectin concentration interaction on cellular phenotypes

Polygenic score	Fibronectin conc.	Area beta	Area SE	Area P	Roundness beta	Roundness SE	Roundness P	Width:length beta	Width:length SE	Width:length P
Cross-psychiatric_0.001_	5	0.0213	0.0130	0.103	−0.0176	0.0144	0.221	−0.0136	0.0104	0.190
Cross-psychiatric_0.001_	25	0.0235	0.0130	0.071	−0.0124	0.0144	0.390	−0.0075	0.0104	0.469
Cross-psychiatric_1_	5	**0.0519**	**0.0150**	**5.61x10**^−^^**4**^	−0.0138	0.0166	0.406	0.0023	0.0119	0.847
Cross-psychiatric_1_	25	0.0218	0.0150	0.147	0.0073	0.0166	0.659	0.0220	0.0119	0.0645
Cross-psychiatric_23andMe_	5	0.0162	0.0135	0.230	−0.0064	0.0149	0.666	0.0007	0.0108	0.945
Cross-psychiatric_23andMe_	25	0.0246	0.0135	0.0691	−0.0014	0.0149	0.923	0.0075	0.0108	0.488
Schizophrenia_0.05_	5	0.0082	0.0127	0.518	−0.0092	0.0141	0.513	−0.0182	0.0101	0.0724
Schizophrenia_0.05_	25	0.0009	0.0127	0.944	−0.0053	0.0141	0.706	−0.0182	0.0101	0.0722
Schizophrenia_1_	5	0.0166	0.0130	0.201	−0.0103	0.0144	0.476	−0.0132	0.0104	0.203
Schizophrenia_1_	25	0.0074	0.0130	0.569	−0.0184	0.0144	0.706	−0.0097	0.0103	0.351
BMI_0.001_	5	−0.0140	0.0157	0.374	0.0002	0.0173	0.992	−0.0134	0.0124	0.280
BMI_0.001_	25	−0.0050	0.0157	0.753	−0.0157	0.0173	0.365	−0.0259	0.0124	0.0370
BMI_1_	5	−0.0299	0.0155	0.0535	0.0006	0.0171	0.973	−0.0166	0.0122	0.174
BMI_1_	25	−0.0304	0.0155	0.0501	−0.0203	0.0171	0.234	−**0.0366**	**0.0122**	**0.00270**
Height_0.001_	5	0.0336	0.0157	0.0327	0.0052	0.0172	0.764	0.0353	0.0125	0.00458
Height_0.001_	25	0.0236	0.0157	0.133	0.0268	0.0173	0.121	**0.0566**	**0.0125**	**5.65x10**^−^^**6**^
Height_1_	5	0.0267	0.0151	0.0782	−0.0017	0.0166	0.918	0.0278	0.0120	0.0203
Height_1_	25	0.0156	0.0152	0.304	0.0199	0.0167	0.231	**0.0495**	**0.0120**	**3.70x10**^−^^**5**^

Polygenic scores are referenced as Trait_Threshold_. Fibronectin concentrations are on the scale of μg/mL, and are compared to a suboptimal concentration of 1 μg/mL. Betas are on the scale of standard deviation changes in phenotype for one standard deviation change in polygenic score at each fibronectin concentration, above and beyond the effect of the polygenic score at the reference fibronectin concentration. Significant associations shown in bold (*p* < 0.00416). All values drawn from multiple linear regressions, full models in Supplementary Table 6

## Discussion

Understanding the biological effects of the polygenic component of complex disorders will be a challenging but necessary step in understanding their aetiology. Human iPSCs offer a potentially valuable model for studying such effects. In this paper, I have shown that applying polygenic scores to databases of iPSCs is feasible but requires overcoming several technical challenges. I discuss each of these challenges below.

The primary challenge is donor number. The power analyses I present show that measuring multiple cells from the same donor provides sufficient power for informative polygenic analyses, despite the small number of donors assessed. The power gain will vary between datasets and phenotypes, dependent on the average correlation between multiple measurements from the same donor (Faes et al. 2009). In these analyses, I estimate that measuring approximately 25,000 cells per donor on average increased the power of polygenic score analyses by a factor of 14–40 relative to the donor number alone. This increase in power is sufficient to provide 80% power for plausible values of both the heritability of the cellular phenotype and of the genetic correlation with complex traits. For example, the Cross-psychiatric phenotype examined in this paper has a genetic correlation with brain putamen volume (SNP-based heritability = 0.36) of 0.2—a cellular phenotype of similar heritability and genetic correlation would be powered for analyses in the HipSci dataset (assuming an effective *N* = 2435) (Cross-Disorder Group of the Psychiatric Genomics Consortium et al. 2019). Currently available iPSC datasets are, therefore, powered for the polygenic exploration of complex trait phenotypes, for cellular phenotypes with a sufficiently strong genetic basis.

The power analyses presented are generalisable to polygenic score analyses examining any repeated measure from the same individual, not just measures of cell morphology. They are also generalisable to other model systems, including iPSC-derived neurons, which are better models for behavioural phenotypes. The genetic covariance between behavioural polygenic scores and neuronal phenotypes is likely to be small, but the power analyses suggest that a dataset of 10 s–100 s of donors, with 1000 s of neurons per donor, would be a practical target for analyses.

The HipSci dataset was intended as a community resource, and its creation required multiple laboratories and considerable time and investment (Streeter et al. 2017). Creating similarly sized and measured datasets will be a major undertaking. However, high-throughput methods for the rapid assessment of cellular phenotypes are being developed. For example, the Census-seq cell village approach allows cells from multiple donors to be plated and assayed together on a single dish (Mitchell et al. 2020). In addition to enabling large-scale cell-level analyses, individual donor genetic associations with cellular phenotypes can be inferred computationally using this method, enabling polygenic score analyses. Building large-scale cell datasets is therefore technically feasible, and further technical developments may reduce the time and costs required.

In this paper, higher Cross-psychiatric_1_ polygenic scores were significantly associated with greater cell area in iPSCs (The association with cell roundness was also sizable, and close to statistical significance). This association was robust to sensitivity analyses, suggesting it was not an artefact of the analytical method. The Cross-psychiatric_1_–fibronectin interaction was also significantly associated with cell area. Fibronectin is an extracellular matrix protein, the concentration of which influences how well the cell can adhere to the plate surface, and therefore affects the cell area (Vigilante et al. 2019). The effect of plating on an optimal concentration of fibronectin (compared to a suboptimal concentration) was greater in cells with a high polygenic score than in cells with a low polygenic score. As such, the polygenic score may (in part) be capturing variability in cell-extracellular matrix adhesion. Cell-extracellular matrix adhesion is fundamental to cell crawling and axon guidance (Letourneau et al. 1992; Walsh and Doherty 1997; Long and Huttner 2019), which has also been implicated in psychiatric disorders (Wray et al. 2018; Cross-Disorder Group of the Psychiatric Genomics Consortium et al. 2019; Trubetskoy et al. 2022; Mullins et al. 2021). The composition of the extracellular matrix, and increased adhesion of cells to the matrix, has been shown to increase neurite extension in neurons (Letourneau et al. 1992; Long and Huttner 2019), to stimulate gyrification in the developing human neocortex (Long et al. 2018), and to promote branching and migration of neurons (Chai et al. 2015; Long and Huttner 2019), among other roles (Long and Huttner 2019).

Variability in cell-extracellular matrix adhesion affecting neuron migration may be one among many general mechanisms influencing the neurobiology of psychiatric disorders. This might explain why there was no significant association between schizophrenia polygenic scores and cell area. There may have been no association with the schizophrenia polygenic score because it is dominated by other (potentially schizophrenia specific) mechanisms. This fits with the results of the latest schizophrenia GWAS from the Psychiatric Genomics Consortium, where genes associated with schizophrenia were not enriched for a role in neuron migration, unlike genes associated with cross-psychiatric disorder risk in the cross-psychiatric disorder GWAS (Cross-Disorder Group of the Psychiatric Genomics Consortium et al. 2019; Trubetskoy et al. 2022). The association with cell area was seen only with the full polygenic score (*p* < 1) for cross-psychiatric disorder risk, not the score limited to variants with stronger evidence for association (*p* < 0.001). One explanation for this may be that individual genetic associations with cross-psychiatric disorder risk that act through effects on cell-extracellular matrix adhesion are likely to be very small. Accordingly, they may not be estimated accurately enough to be enriched in the more limited score from the cross-psychiatric disorder GWAS, and so the association between cross-psychiatric disorder risk and cell area is only observed in the full score (Cross-Disorder Group of the Psychiatric Genomics Consortium et al. 2019; Sullivan and Geschwind 2019).

Even when only considering the baseline fibronectin concentration (1 μg/mL), there was still an effect of the cross-psychiatric disorder polygenic score on cell area, suggesting the effect cannot be purely driven by cell–extracellular matrix interactions. Other possibilities may include an effect on cell morphogenic processes within the cell, such as altering the action or composition of the cytoskeleton (Courtot et al. 2014; Närvä et al. 2017). However, given that the association between the cross-psychiatric disorder polygenic score and cell area is close to the threshold for statistical significance, I cannot conclude strongly it is stable and generalisable. As sufficiently sized iPSC datasets emerge, it would be of interest to replicate this finding.

Certain limitations of this work should be taken into account. Foremost among these is that iPSCs are not in vivo cells, but rather in vitro models. As such, they are abstracted from the biological context of in vivo cells, and are affected by the environment of the laboratory and of cell culture in a way that in vivo cells are not affected. However, previous analyses in this dataset have shown that iPSC morphology is affected both by extrinsic factors (such as fibronectin concentration), and by factors intrinsic to the cell (Vigilante et al. 2019). These intrinsic factors are likely reflect to molecular mechanisms of cell shape more generally, which would apply to cells within their in vivo context. Furthermore, iPSC cells share the genome of their donor, and as such reflect genetic variation between donors. If genetic variation between donors is correlated with iPSC cell phenotypic variation, this supports a role for genetic variation in molecular mechanisms affecting cell morphology in general.

A further limitation is that broad phenotypic information on the donors was not available. As such, I cannot exclude that donors may have been phenotypic outliers for the polygenic score traits. For example, if a donor had schizophrenia, they may have a higher polygenic score for psychiatric disorders than the population average. Similarly, because the donors are anonymous, it is not known whether they contributed to the GWAS from which the polygenic scores were derived. Sample overlap would severely bias the results of the study (Choi et al. 2020). However, the results were stable in leave-one-donor out analyses, suggesting no single donor (such as one that was a phenotypic outlier, or who might have participated in one of the GWAS) strongly influenced the results. An unmitigated limitation is that the study was restricted only to donors of European ancestries, due to the availability of cell data. Results from polygenic scores are often poorly translated across different ancestries, which limits the broader generalisability of these findings (Martin et al. 2019). Including donors from diverse ancestries should be a key consideration in generating new cellular datasets.

In summary, I have demonstrated that polygenic score analysis is feasible in existing datasets of iPSCs, and that this holds considerable promise for examining the polygenic effects of complex disorders in cellular models. Emergent cell biology techniques such as the Census-seq approach provide a mechanism to achieve the sample sizes needed for the analyses described in this paper. Coupled with further advances in the derivation of diverse cell types from iPSCs, polygenic scoring in cell lines has the potential to be a powerful technique for the assessment of cellular proxies of complex traits.

## Supplementary Information

Below is the link to the electronic supplementary material.

Supplementary file1 (DOCX 2229 KB)

Supplementary file2 (XLSX 338 KB)

## Data Availability

All data analysed are available via the Human-Induced Pluripotent Stem Cell Initiative project website http://www.hipsci.org/. Imaging data are available open access from the HipSci website. Genotype data are available under controlled access (accessed under a data transfer agreement with the Wellcome Sanger Institute) and open access conditions. Controlled access data are hosted at the European Genome-phenome Archive at the European Bioinformatics Institute under accession number EGAD00010001147. Open-access genotypes are hosted at the European Nucleotide Archive at the European Bioinformatics Institute under project ID PRJEB11750. Summary statistics were downloaded from the GIANT consortium (https://portals.broadinstitute.org/collaboration/giant/index.php/GIANT_consortium_data_files), the Walters group (https://walters.psycm.cf.ac.uk/), and the Psychiatric Genomics Consortium (https://www.med.unc.edu/pgc/download-results/cd/).

## Code availability

Code underlying the analyses presented is available at https://github.com/ColemanResearchGroup/hipsciPGS.
