# Supplementary material for: Feasibility and application of polygenic score analysis to the morphology of human-induced pluripotent stem cells
Source: Mol Genet Genomics. 2022 May 28;297(4):1111–22. doi: 10.1007/s00438-022-01905-2 (PMC9250464; doi:10.1007/s00438-022-01905-2)
Supplement: Supplementary file 1 — Supplementary file1 (DOCX 2229 KB) [file 438_2022_1905_MOESM1_ESM.docx]

**Supplementary Material for "Feasibility and application of polygenic score analysis to the morphology of human induced pluripotent stem cells"**

Author: Jonathan R. I. Coleman^1, 2, https://orcid.org/0000-0002-6759-0944, *^

^1^Social, Genetic and Developmental Psychiatry Centre, Institute of Psychiatry, Psychology and Neuroscience, King's College London, UK

^2^National Institute for Health Research Maudsley Biomedical Research Centre, South London and Maudsley NHS Foundation Trust, London, UK

*Address correspondence to Jonathan Coleman, SGDP Centre, IoPPN - PO80, DeCrespigny Park, Denmark Hill, London, UK, SE5 8AF. jonathan.coleman@kcl.ac.uk

[Supplementary Methods 1](#_Toc68871941)

[Cellular phenotyping 1](#_Toc68871942)

[Genome-wide genotype data quality control and imputation 1](#_Toc68871943)

[Cell lines from the same donor were genetically identical 2](#_Toc68871944)

[Polygenic score power analyses, and estimation of effective N 3](#_Toc68871945)

[Supplementary Results 4](#_Toc68871946)

[Power analyses 4](#_Toc68871947)

[Schizophrenia 4](#_Toc68871948)

[Body mass index 5](#_Toc68871949)

[Height 5](#_Toc68871950)

[Sensitivity analyses: leave-one-donor-out models 6](#_Toc68871951)

[Sensitivity analyses: number of genomic principal components 6](#_Toc68871952)

[Sensitivity analyses: coding of cells in clump 7](#_Toc68871953)

[Supplementary Figures 8](#_Toc68871954)

[Supplementary Figure 1 8](#_Toc68871955)

[Supplementary Figure 2 9](#_Toc68871956)

[Supplementary Figure 3 10](#_Toc68871957)

[Supplementary Figure 4 11](#_Toc68871958)

[Supplementary References 12](#_Toc68871959)

# Supplementary Methods

## Cellular phenotyping

Induced pluripotent stem cells (iPSCs) were generated from fibroblasts or peripheral blood mononuclear cells via episomal DNA or Sendai vector transduction (Vigilante et al 2019). IPSCs were cultured in a feeder (mouse embryonic fibroblasts) or feeder-free environment, before assessment on a fibronectin adhesion assay (Vigilante et al 2019). From each cell line, 3000 iPSCs were plated for 24 hours on a single well coated with varying concentrations of the extracellular matrix protein fibronectin: 1μg/mL (sub-optimal for cell adhesion), 5μg/mL and 25μg/mL (Vigilante et al 2019). For each cell line, each condition was performed in triplicate to mitigate well edge and position effects (Vigilante et al 2019). Single IPSCs, alone or aggregated in clumps of cells, were imaged using an Operetta (Perkin Elmer) high content device, and processed, quantified, and normalised as previously described (Leha et al 2016).

## Genome-wide genotype data quality control and imputation

Genome-wide genotype data was available for all analysed cell lines from the Illumina HumanCoreExome-12 v1 BeadChip, previously imputed to a combined 1000 Genomes and UK10K reference panel (Kilpinen et al 2017). I initially included data from 280 cell lines (including source cells – 60 fibroblasts and 5 peripheral blood mononuclear cells – and 215 iPSC lines) from 60 donors with cellular phenotype data. Data underwent quality control in PLINK 1.9 and R 3.6 (Core Team and Others 2013; Chang et al 2015; Coleman et al 2016). For genotype quality control and the generation of polygenic risk scores, I retained a single cell-line for each donor (as cell lines from the same donor were approximately genetically identical – see below). I excluded variants with minor allele frequency (MAF) <0.05, call rate <0.99, and which were outside of Hardy-Weinberg equilibrium (HWE p-value < 10^-5^). Following this step, I limited variants to those present on the genotyping array only, and re-imputed the data on the Sanger imputation server, imputing to the Haplotype Reference Consortium panel release 1.1, using the PBWT + Eagle pipeline (Durbin 2014; McCarthy et al 2016; Loh et al 2016). I retained variants from the HRC-imputed data with imputation INFO ≥0.9, and MAF ≥0.05. I plotted cell line data on principal components of genome-wide genotype data from 1000 Genomes project participants (1000 Genomes Project Consortium et al 2015). This confirmed all cell lines were from donors from European ancestries. I also generated principal components from the genome-wide genotype data of the donors for use as covariates in analysis. Reported sex of donors was consistent with the heterozygosity of X chromosome variants in the cell lines. All donors were unrelated (pi-hat <0.125) and well-genotyped (call rate ≥99%).

## Cell lines from the same donor were genetically identical

I limited initial genotype data to polymorphic variants (MAF ≥0.001) and generated call rates per cell line using PLINK 1.9. All call rates were very high (≥99%), both when considering only genotyped variants and when also considering imputed variants in the combined 1000 Genomes and UK10K reference panel. I then estimated identical-by-descent values between all cell lines in the data using the *--genome* function in PLINK 1.9. All cell lines were highly concordant (minimum IBD pi-hat estimate 0.984) and consistent with typical values obtained by genotyping duplicates of the same sample. As such, cell lines could be treated as genetically identical, and polygenic scores considered as donor-level variables.

## The calculation of the proportion of variance

The proportion of variance (also referred to as the coefficient of determination) is a standard measure of the fit of data to the line of regression in linear regression. The proportion of variance explained by a polygenic score is calculated as:

$$1- \frac{1-R_{full}^{2}}{1-R_{null}^{2}}$$

where $R_{full}^{2}$ is the proportion of variance explained by the full model including the polygenic score and all covariates, and $R_{null}^{2}$ is the proportion of variance explained by the model without the polygenic score, but with all other covariates.

Residual variance is not well defined on the binary observed scale (Nagelkerke 1991). Therefore, when using logistic regression to assess binary outcomes such as schizophrenia, an approximation of the proportion of variance (referred to as a pseudo measure) is used. The pseudo measure used by PRSice is defined below, and approximates the proportion of variance on the liability scale, making it comparable with the proportion of variance estimated from linear regression (Lee et al 2012):

$$R_{l}^{2}= \frac{R_{OCC}^{2} C}{1+ R_{OCC}^{2} \Theta C}$$

$$C=\frac{K(1-K)}{z^{2}} \times\frac{K(1-K)}{P(1-P)}$$

$$\Theta= m\frac{P-K}{1-K}(m\frac{P-K}{1-K}-t)$$

where $R_{l}^{2}$ is the approximation of the equivalent proportion of variance on the liability scale, and $R_{OCC}^{2}$ is the proportion of variance on the observed scale from a linear regression of a binary outcome. $K$ is the prevalence of the disease in the population, $z$ is the height of the normal density curve at $K$, and $P$ is the proportion of disease cases in the sample. $m$ is the mean liability of disease cases, and $t$ is the threshold on the normal distribution that truncates the proportion of disease prevalence $K$ (Lee et al 2012).

## Polygenic score power analyses, and estimation of effective N

I calculated power for polygenic score analyses using the AVENGEME method (Palla and Dudbridge 2015). The underlying equations describing these analyses are defined below, adapted from Dudbridge (2013), for the linear additive genetic effect of a polygenic score ($\hat{S})$ derived from a quantitative phenotype ($Y_{1})$ on a second quantitative phenotype ($Y_{2})$:

$Y_{1}= \sum_{i=1}^{m} \beta_{i1}G_{i1}+ E_{1}, Y_{2}= \sum_{i=1}^{m} \beta_{i2}G_{i2}+ E_{2}$,

$Y_{2}\sim\hat{S}=\sum_{i=1}^{m} \hat{\beta}_{i1}G_{i2}$,

$\lambda=\frac{N_{2}R_{\hat{S},Y_{2}}^{2}}{(1- R_{\hat{S},Y_{2}}^{2})}$,

$R_{\hat{S},Y_{2}}^{2}= \frac{m {cov(\hat{\beta}_{i1},\beta_{i2})}^{2}}{var(\hat{\beta}_{i1})var(\beta_{i2})}$,

$Power=1- \Phi\left( \Phi^{-1}\left( 1- \frac{\alpha}{2} \right)-\sqrt{\lambda} \right)+ \Phi\left( \Phi^{-1}\left( \frac{\alpha}{2} \right)-\sqrt{\lambda} \right)$

$Y_{j}$ can be expressed as the linear combination of each variant $G_{ij}$ with coefficient $\beta_{ij}$, as well as the error term $E_{j}$ (independent of $\beta_{ij}$), for $m$ variants. A polygenic score ($\hat{S}$) can be created from $\hat{\beta}_{ij^{'}}$ (the estimated effect of variant $G_{i}$ on phenotype $Y_{j^{'}}$). Note that $\hat{S}$ is comprised of the estimated effects of each variant $G_{i2}$ on phenotype $Y_{1}$ – that is, the polygenic score is constructed from effects on phenotype 1, but is assessed in relation to the genotypes and phenotype from study 2. The non-centrality parameter of the $\chi_{1}^{2}$ test of association between $Y_{2}$ and $\hat{S}$ is labelled $\lambda$, and relies on $N_{2}$ (the sample size of the analysis of $Y_{2}$) and on $R_{\hat{S},Y_{2}}^{2}$ (the variance explained by $\hat{S}$ on $Y_{2}$), which in turn relies on $m,$on the covariance of $\hat{\beta}_{i1}$(the estimated effect of variant $G_{i1}$ on phenotype $Y_{1}$) with $\beta_{i2}$ (the true effect of variant $G_{i2}$ on phenotype $Y_{2}$), and on the product of the variances of $\hat{\beta}_{i1}$ and $\beta_{i2}.$ The power of the $\chi_{1}^{2}$ test of association between $Y_{2}$ and $\hat{S}$is dependent on the non-centrality parameter, as well as the significance threshold ($\alpha)$. Note that $\Phi$in the power equation denotes the cumulative distribution function of the normal distribution.

In these analyses, I defined $Y_{1}$ as the phenotype studied in each base GWAS, and $Y_{2}$ as some hypothetical cellular phenotype in the target dataset. I then examined the association of a polygenic score calculated from the base GWAS ($\hat{S})$with this cellular phenotype. I estimated what value of genetic covariance ($cov(\hat{\beta}_{i1},\beta_{i2})$) would result in a large enough non-centrality parameter that the associated $\chi_{1}^{2}$ test has ≥80% power at $\alpha=0.05$. I used fixed values of $\hat{\beta}_{i1}$ (that is, the common genetic contribution to variance from the polygenic score applied to the GWAS phenotype, which is known from the GWAS) and varied both the value of the genetic covariance, and the value of $\beta_{i2}$. For ease of interpretation, I converted genetic covariance to genetic correlation (as described in the main text), for each pair of values for genetic covariance, and $\beta_{i2}$.

In the initial polygenic score power analyses, I assumed $N_{2}$=60 (the number of donors). This is conservative, because it assumes that all observations from the same donor are perfectly correlated, and therefore that there is no benefit of having multiple phenotypic observations for the same donor. This assumption is likely to be voided in practice. Non-independent observations alter the standard calculation of sample variance from $\sigma^{2}/ N$ to $\sigma^{2}/ N_{eff}$, where $\sigma$ is the population standard deviation, $N$ is the number of observations, and $N_{eff}$ is the effective number of independent observations. Assuming observations between donors are uncorrelated, $N_{eff}$ can be described as follows (Faes et al 2009) :

$N_{eff}=\Sigma\frac{n_{i}}{1 + \rho(n_{i}-1)}$

where $n_{i}$ is the size of each set of observations from the same donor, and $\rho$ is the correlation between observations from the same donor, calculated from the random components of variance attributable to the random effects ($\tau^{2}$) and the residuals ($\sigma^{2}$):

$\rho= \frac{\tau^{2}}{(\tau^{2} + \sigma^{2})}$

For the data presented in the main text, each value of $n_{i}$ is known (Supplementary Table 2), and $\tau^{2}$ and the residuals $\sigma^{2}$ are estimated for each analysis as the random effect of donor, and the sum of the random effect of well and the residual respectively (Supplementary Tables 5). As such, we can approximate $N_{eff}$ from the analyses to assess the decrease in power from assuming that all observations from the same donor are perfectly correlated. The minimum value of $\rho$ observed in the data was 0.025 (analysis of PGS BMI_1_ effects on cell width-to-length ratio), which results in $N_{eff}$ = 2435. In comparison, the maximum value was 0.071 (analysis of PGS SCZ_0.05_ effects on cell roundness), with $N_{eff}$ = 850 respectively.

# Supplementary Results

## Power analyses

### Schizophrenia

At the current donor number (n = 60), analyses have ≥80% power only when the genetic covariance between Schizophrenia_1_ and the cellular phenotype is ≥0.28. This could correspond to a genetic correlation ≥0.8 when the cellular phenotype has a SNP-based heritability ≥0.55 (Supplementary Figure 2a, Supplementary Table 3).

Accounting for the power gain from measuring multiple cells from the same donor (Neff = 850-2435), analyses have ≥80% power when the genetic covariance between Schizophrenia_1_ and the cellular phenotype is ≥0.05-0.08. This corresponds to (for example) a genetic correlation ≥0.15-0.25 when the cellular phenotype has a SNP-based heritability ≥0.55, or a genetic correlation ≥0.35-0.55 when the SNP-based heritability is ≥0.1 (Supplementary Figures 2b, 2c, Supplementary Table 3).

### Body mass index

At the current donor number (n = 60), analyses have ≥80% power only when the genetic covariance between BMI_1_ and the cellular phenotype is ≥0.16. This could correspond to a genetic correlation ≥0.6 when the cellular phenotype has a SNP-based heritability ≥0.55 (Supplementary Figure 3a, Supplementary Table 3).

Accounting for the power gain from measuring multiple cells from the same donor (Neff = 850-2435), analyses have ≥80% power when the genetic covariance between BMI_1_ and the cellular phenotype is ≥0.03-0.05. This corresponds to (for example) a genetic correlation ≥0.1-0.2 when the cellular phenotype has a SNP-based heritability ≥0.55, or a genetic correlation ≥0.25-0.4 when the SNP-based heritability is ≥0.1 (Supplementary Figures 3b, 3c, Supplementary Table 3).

### Height

At the current donor number (n = 60), analyses have ≥80% power only when the genetic covariance between Height_1_ and the cellular phenotype is ≥0.23. This could correspond to a genetic correlation ≥0.55 when the cellular phenotype has a SNP-based heritability ≥0.55 (Supplementary Figure 4a, Supplementary Table 3).

Accounting for the power gain from measuring multiple cells from the same donor (Neff = 850-2435), analyses have ≥80% power when the genetic covariance between Height_1_ and the cellular phenotype is ≥0.04-0.06. This corresponds to (for example) a genetic correlation ≥0.1-0.15 when the cellular phenotype has a SNP-based heritability is ≥0.55, or a genetic correlation ≥0.25-0.35 when the SNP-based heritability is ≥0.1 (Supplementary Figures 4b, 4c, Supplementary Table 3).

## Sensitivity analyses: leave-one-donor-out models

To assess the contribution of individual donors to the association, I ran leave-one-donor-out models for all models without interaction terms (Supplementary Table 7). Estimates of the polygenic score association with each phenotype did not differ substantially from the estimate observed in the main analysis. All estimates from leave-one-donor-out analyses lay within the 95% confidence interval of the observed estimate. The observed estimate was approximately central to the distribution of the leave-one-donor-out estimates. The maximum difference between any observed estimate and the midpoint of relevant leave-one-donor-out estimates was for the association of BMI_1_ with cell width-to-length ratio, where the difference was 16.5% of the observed standard error (observed beta = -0.0377, midpoint = -0.0414). The results of this analysis indicate that no individual donor had a substantial influence on the results of the main analysis.

## Sensitivity analyses: number of genomic principal components

To assess the importance of including genomic principal components on the statistically significant finding reported, I varied the number of genomic principal components included in the model. I compared models with two principal components and ten principal components to the main model with four principal components. This did not significantly alter the result. In the model with two principal components, the effect size of the association of Cross-psychiatric_1_ with cell area was 0.0720, which did not differ from the effect size in the main analysis (main analysis beta = 0.0845, difference p=0.751, two-sample Z test). In the model with ten principal components, the effect size was 0.0808, which also did not differ (p=0.925, two-sample Z test).

## Sensitivity analyses: coding of cells in clump

To assess the importance of coding choices made on the statistically significant finding reported, I altered the coding of the number of cells in each clump. Specifically, I coded this as an ordinal variable (1 cell [single, reference], 2 or 3 cells [multiple, no cells surrounded by other cells], 4 or more cells [multiple, cells surrounded by other cells]), rather than a continuous variable. This did not significantly alter the result: coding clump size ordinally, the effect size of the association of Cross-psychiatric_1_ with cell area was 0.0836, which did not differ from the effect size in the main analysis (p=0.982, two-sample Z test).

# Supplementary Figures

## Supplementary Figure 1


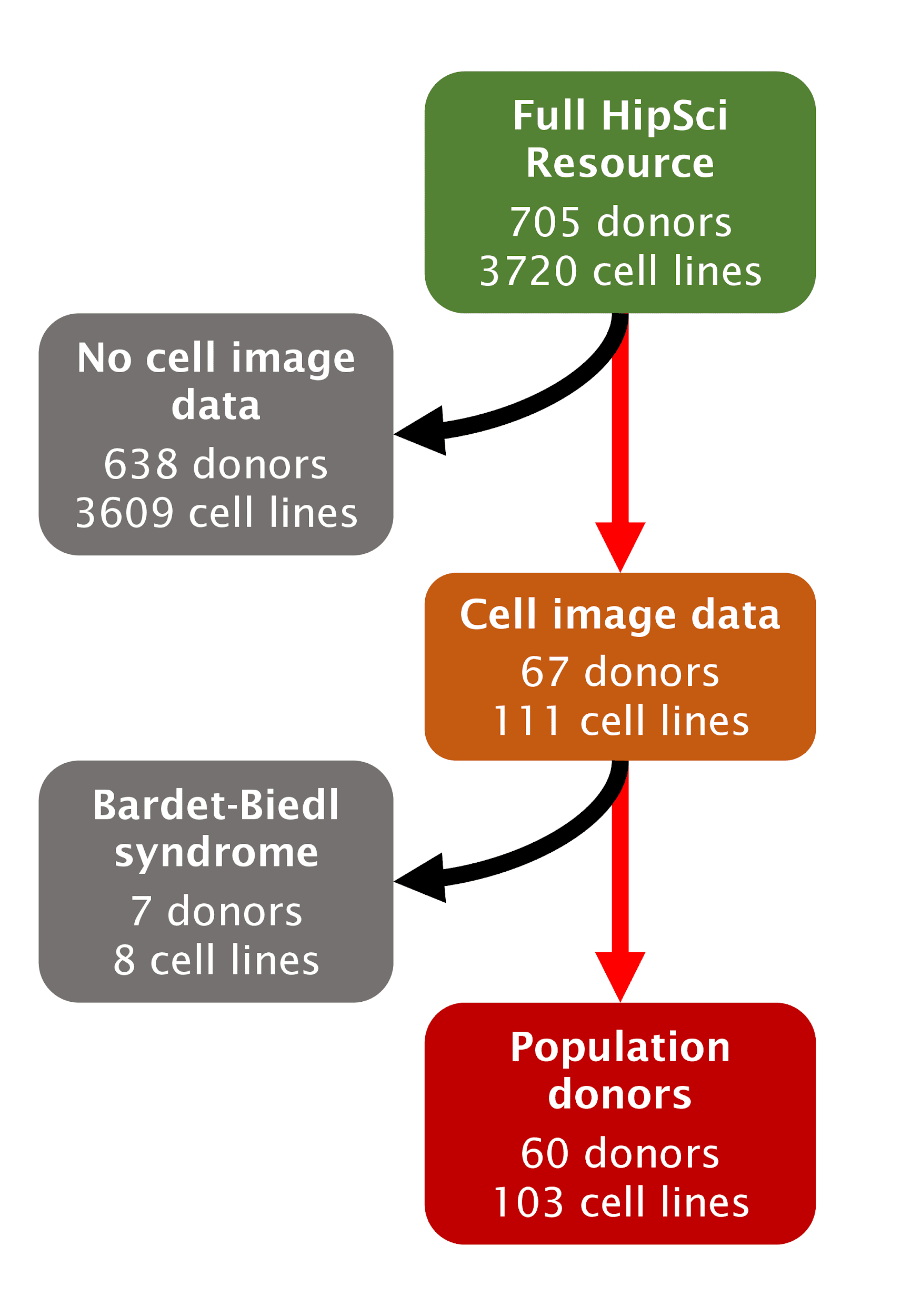


Supplementary Figure 1: Flowchart of donor and cell line inclusion in the analyses. Abbreviations - HipSci = Human Induced Pluripotent Stem Cell Initiative.

## Supplementary Figure 2

**a)**

**
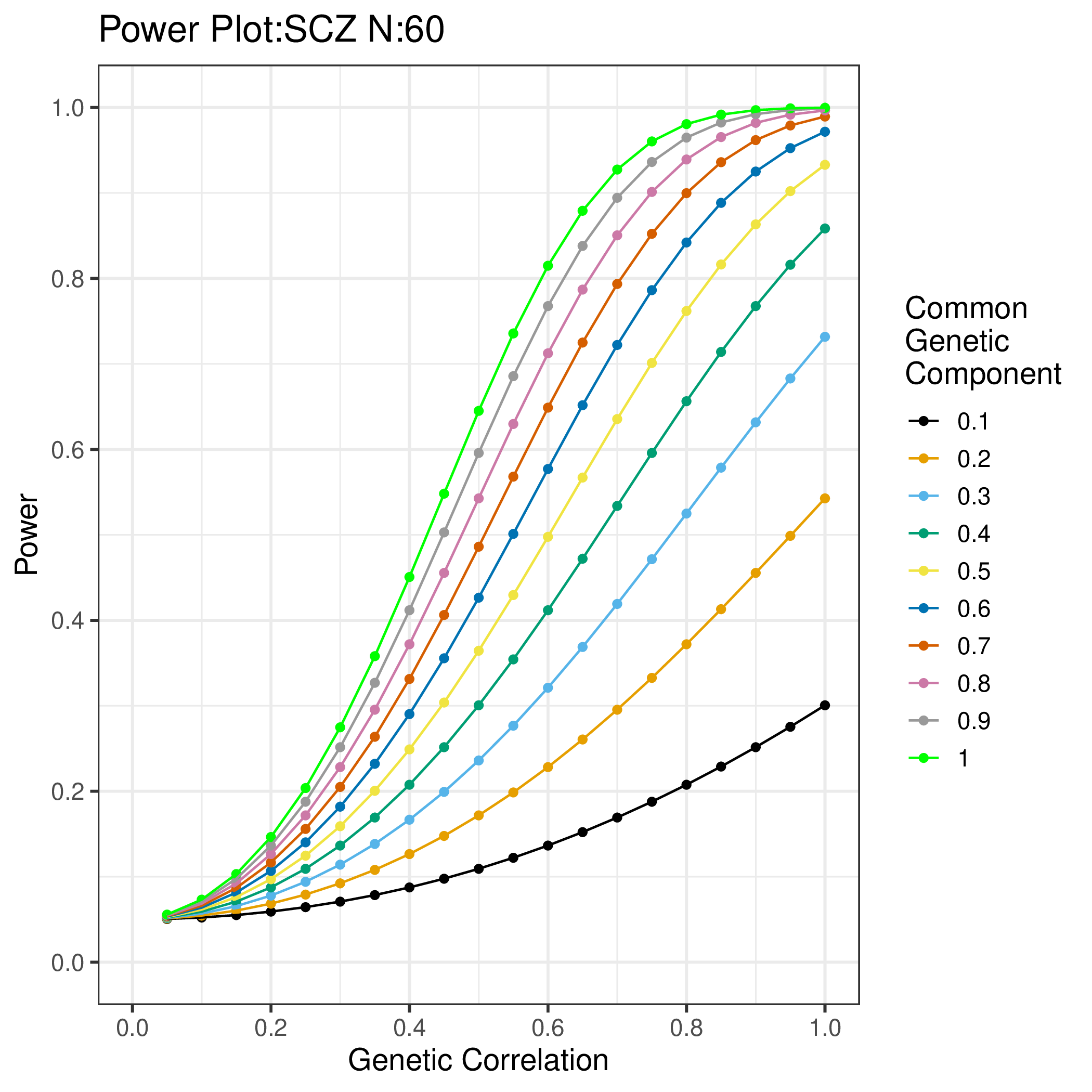
**

**b) c)**

**
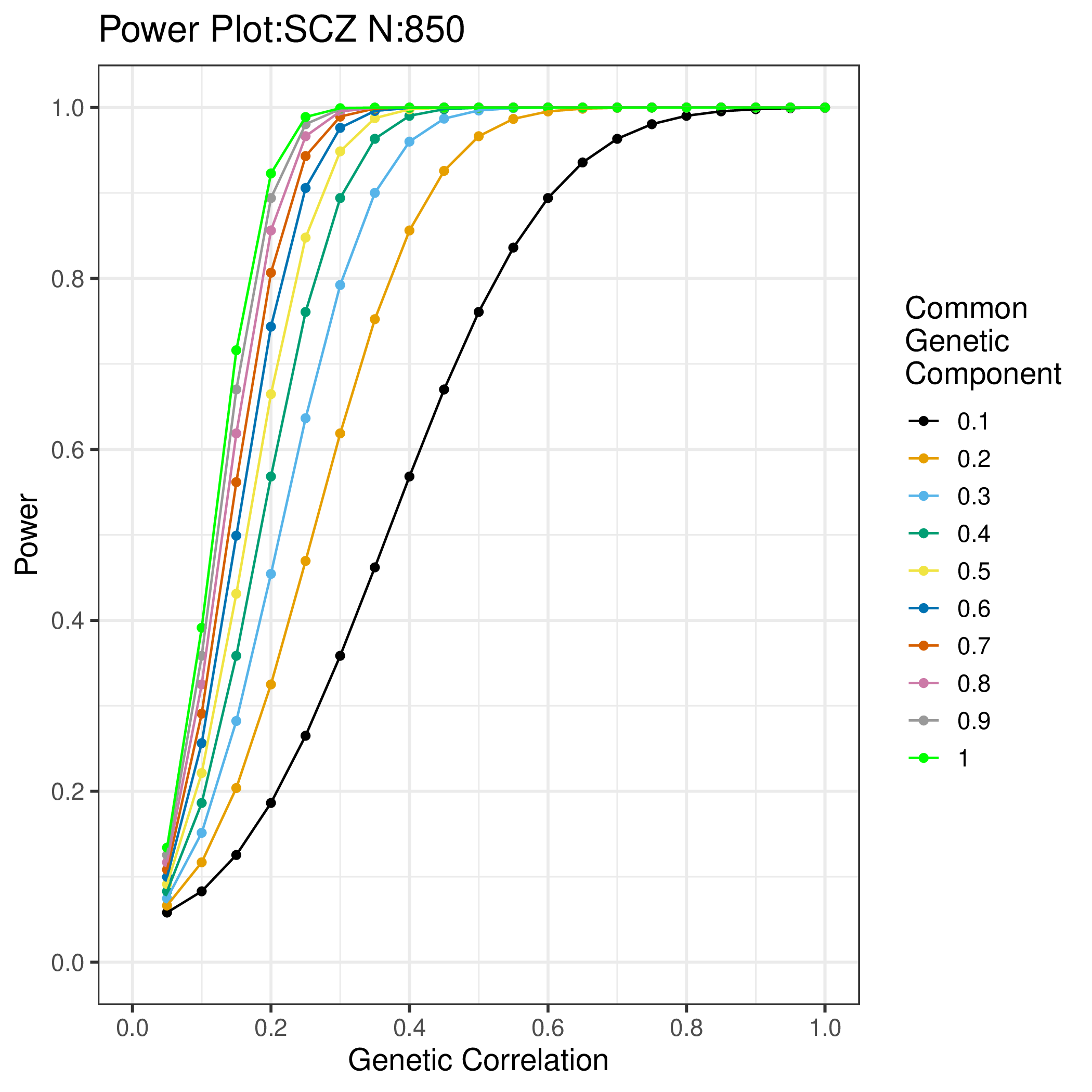

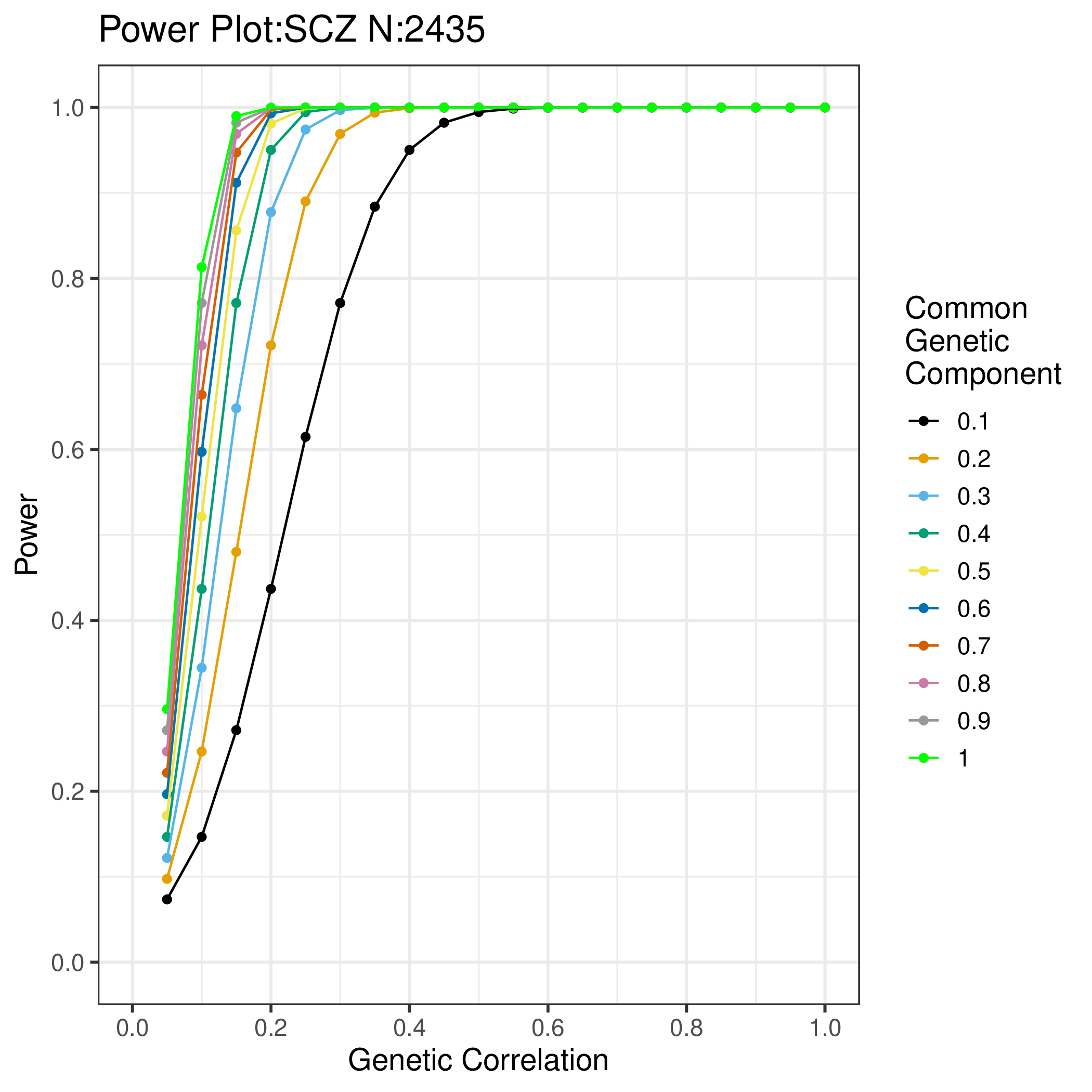
**

Supplementary Figure 2: Power (y axis) to detect a genetic relationship between Schizophrenia_1_ and a cellular phenotype with a common genetic component of varying size (coloured lines) at different values of genetic correlation (x-axis), for differing values of N:

a) 60 [not accounting for multiple measurements],

b) 850 [lower estimate of effective N],

c) 2435 [higher estimate of effective N].

## Supplementary Figure 3

**a)**

**
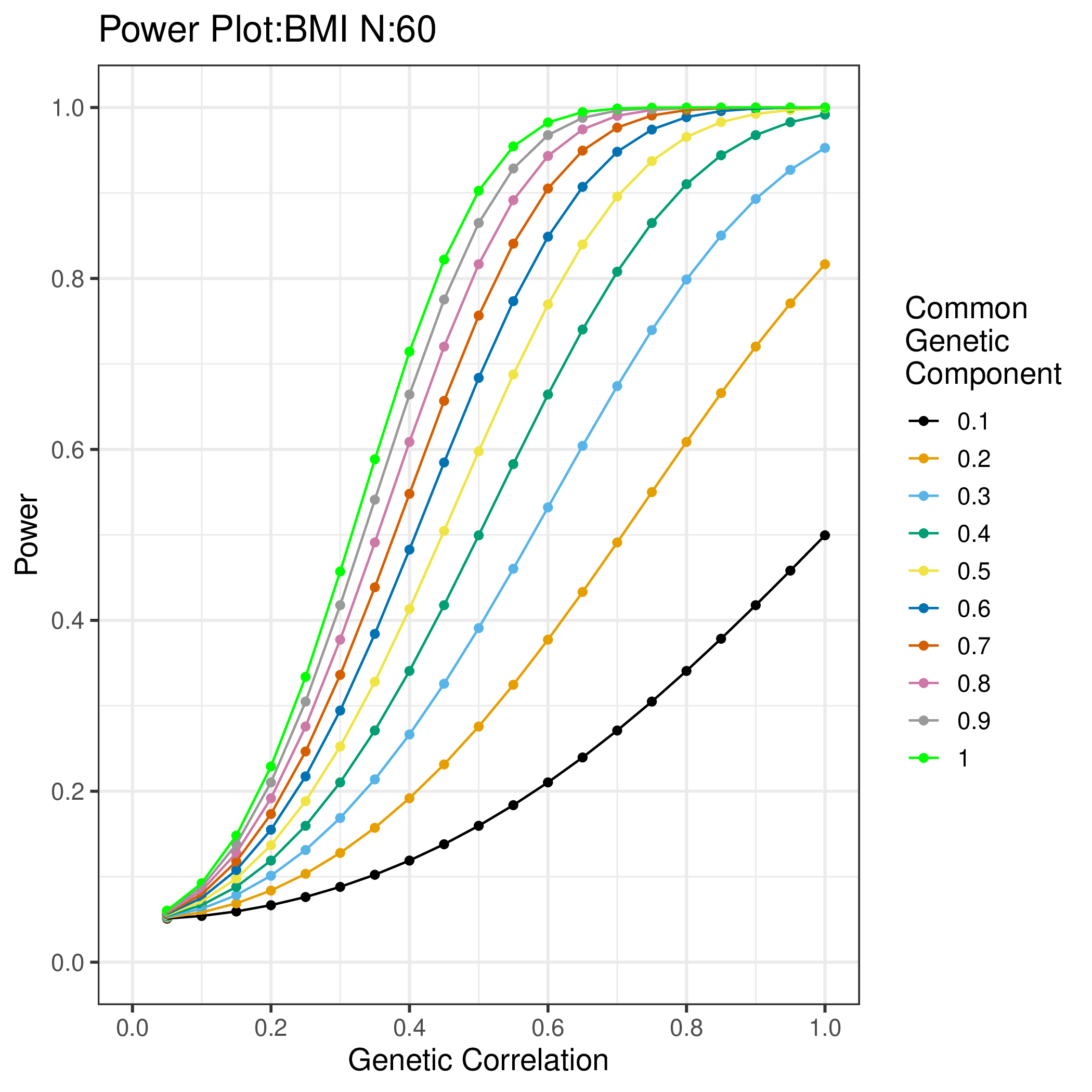
**

**b) c)**

**
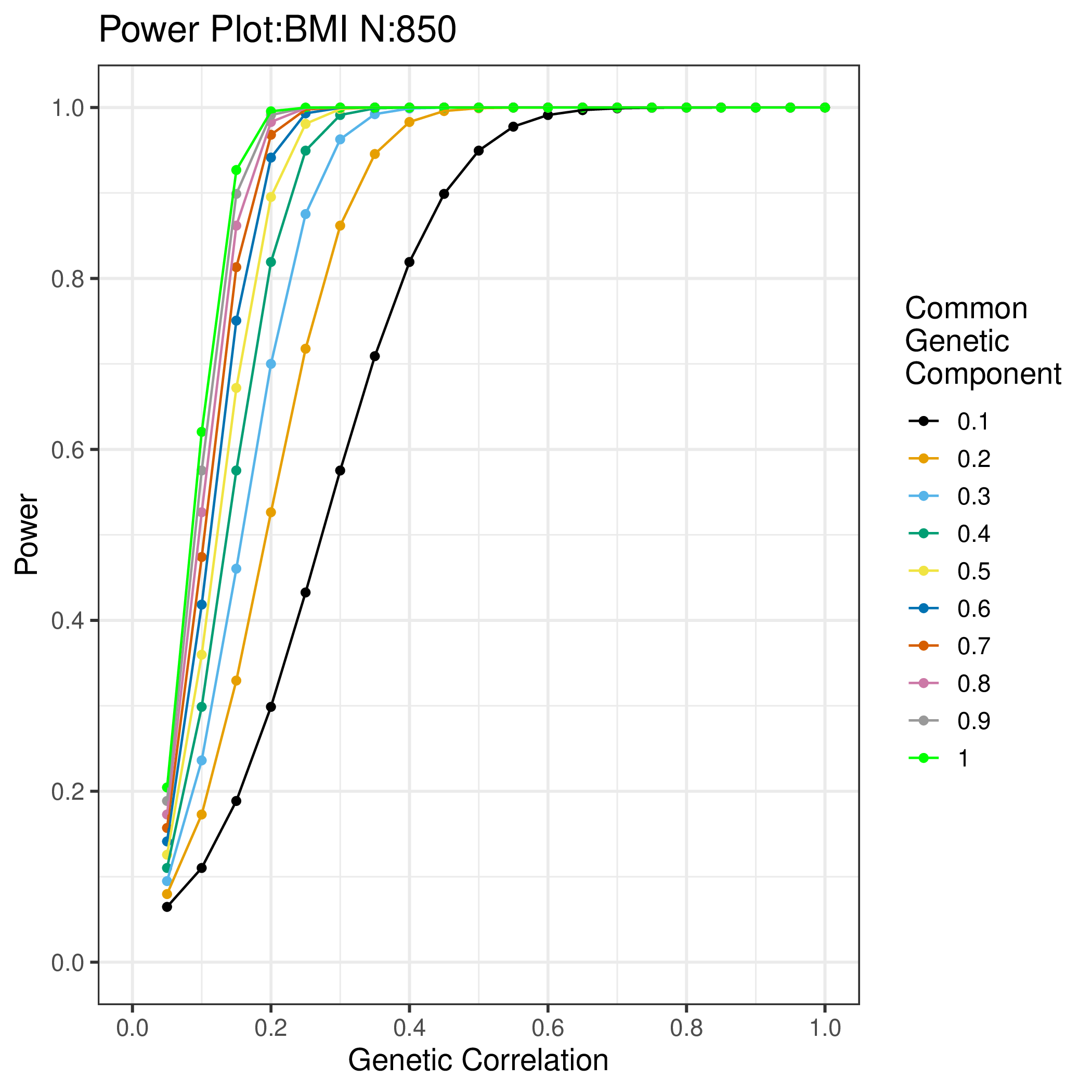

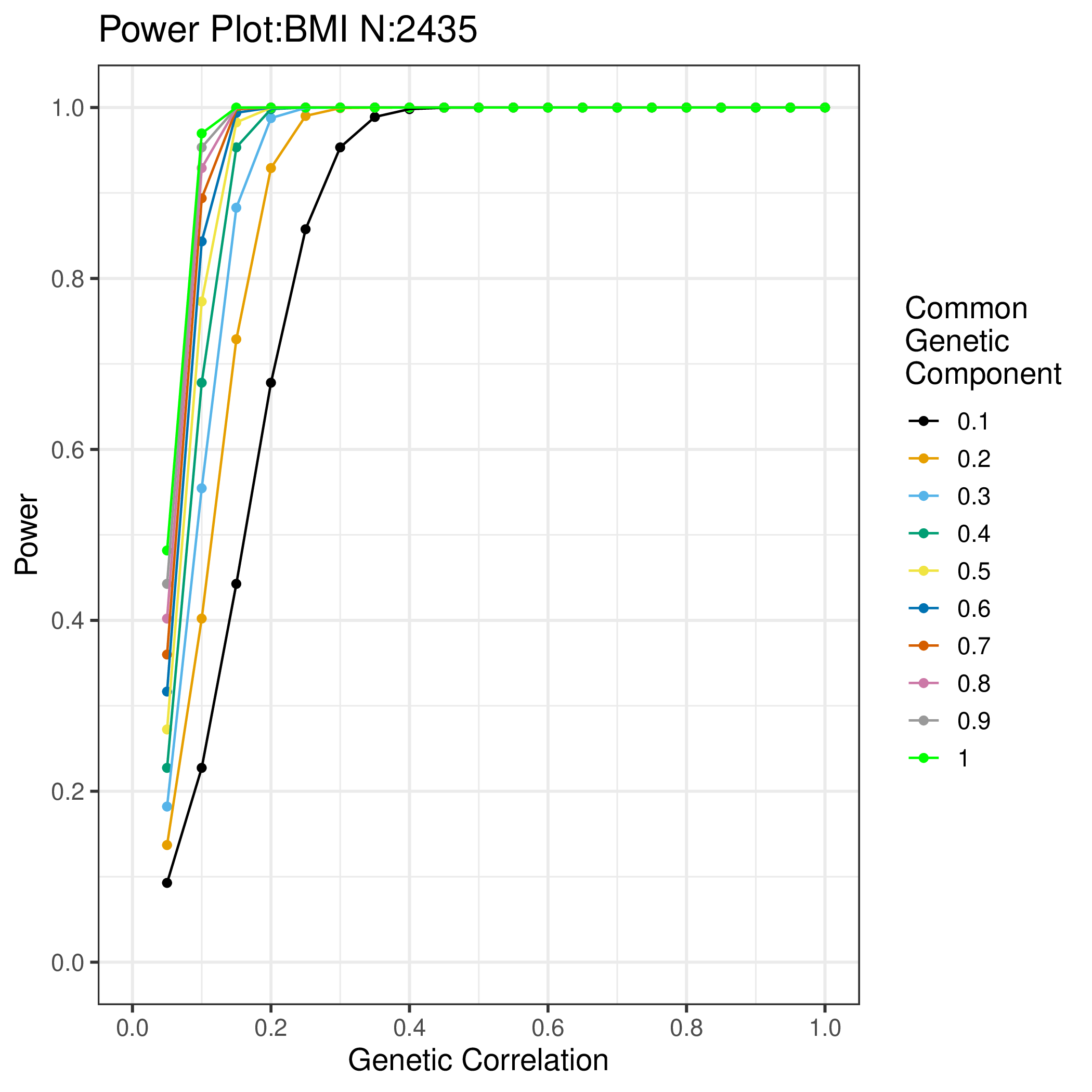
**

Supplementary Figure 3: Power (y axis) to detect a genetic relationship between BMI_1_ and a cellular phenotype with a common genetic component of varying size (coloured lines) at different values of genetic correlation (x-axis), for differing values of N:

a) 60 [not accounting for multiple measurements],

b) 850 [lower estimate of effective N],

c) 2435 [higher estimate of effective N].

## Supplementary Figure 4

**a)**

**
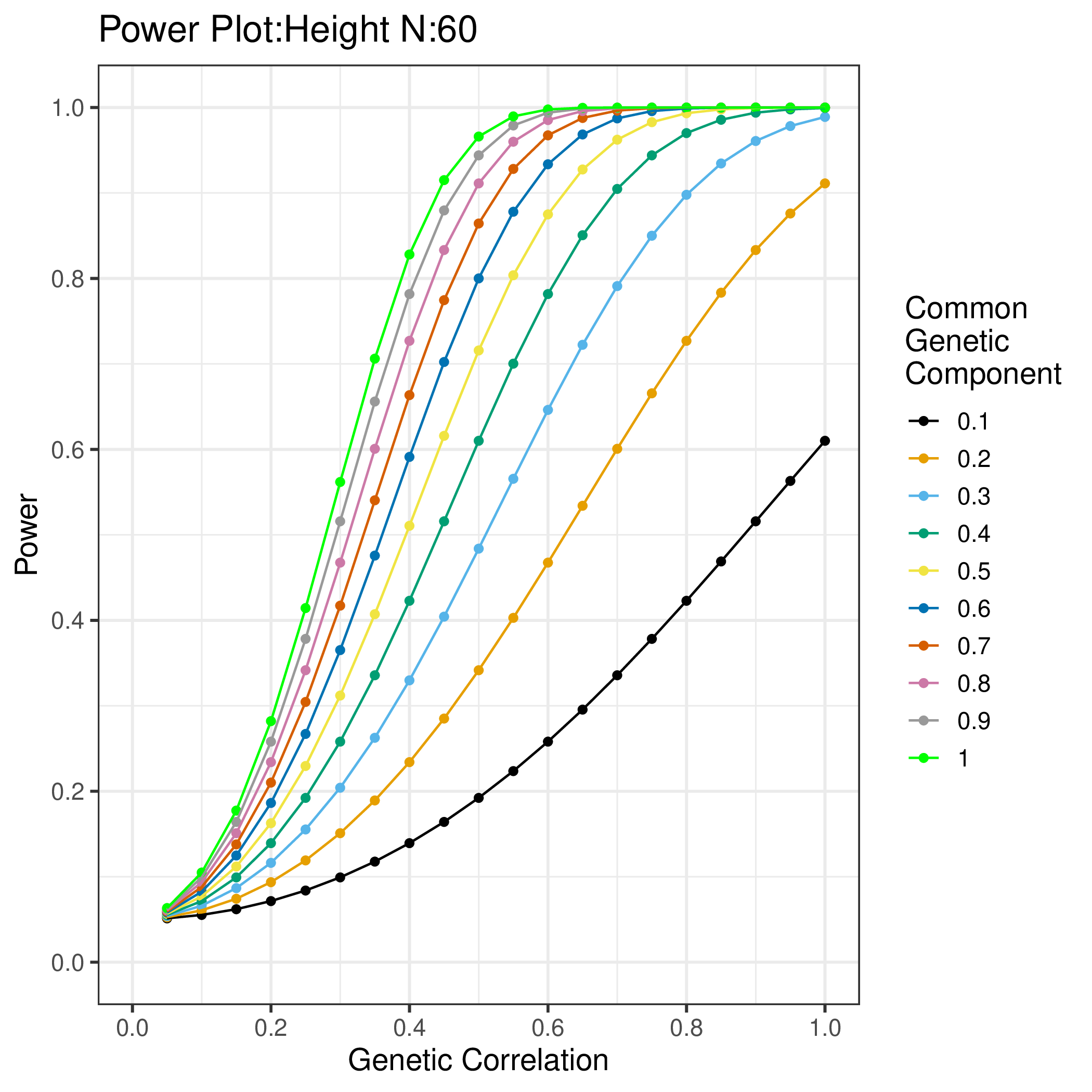
**

**b) c)**

**
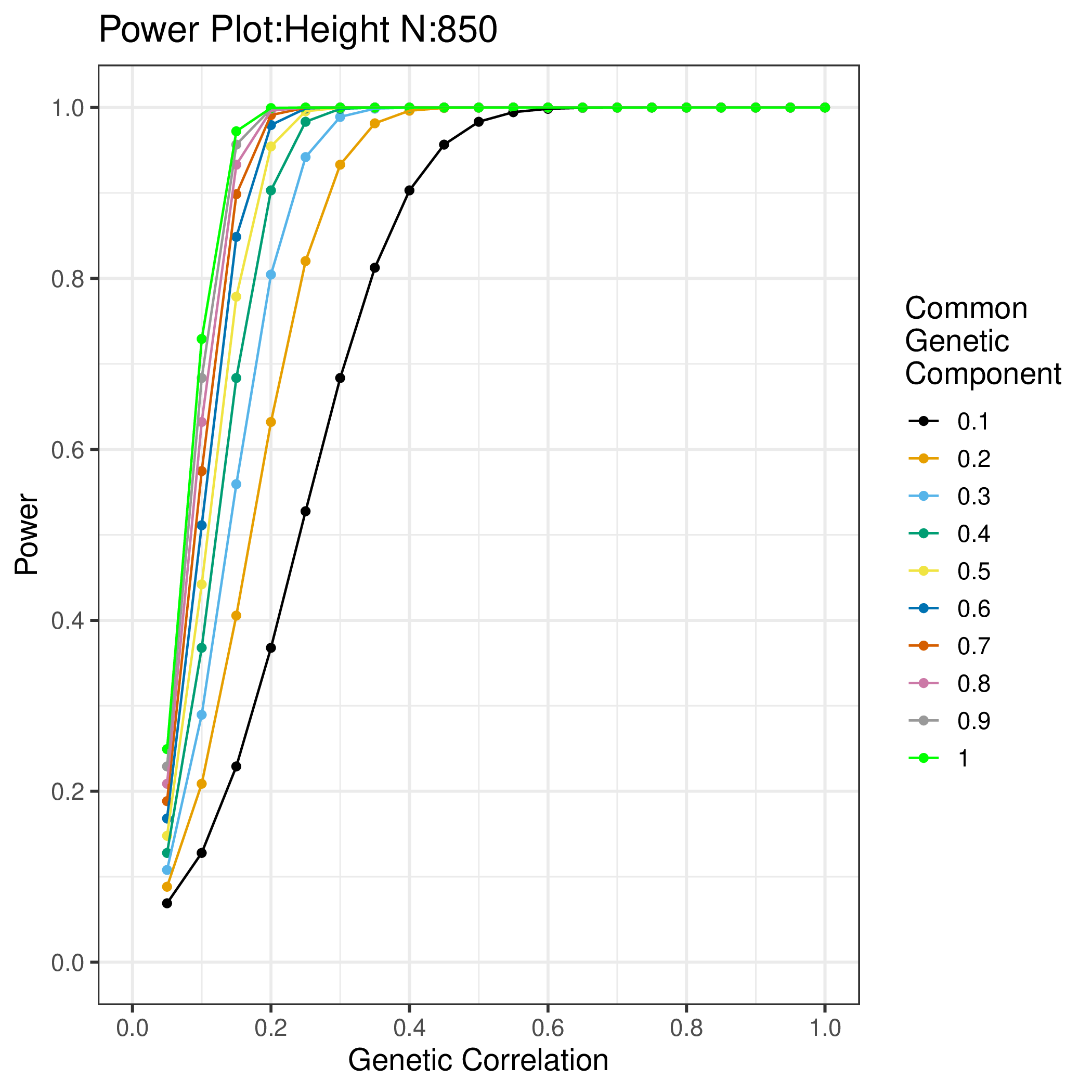

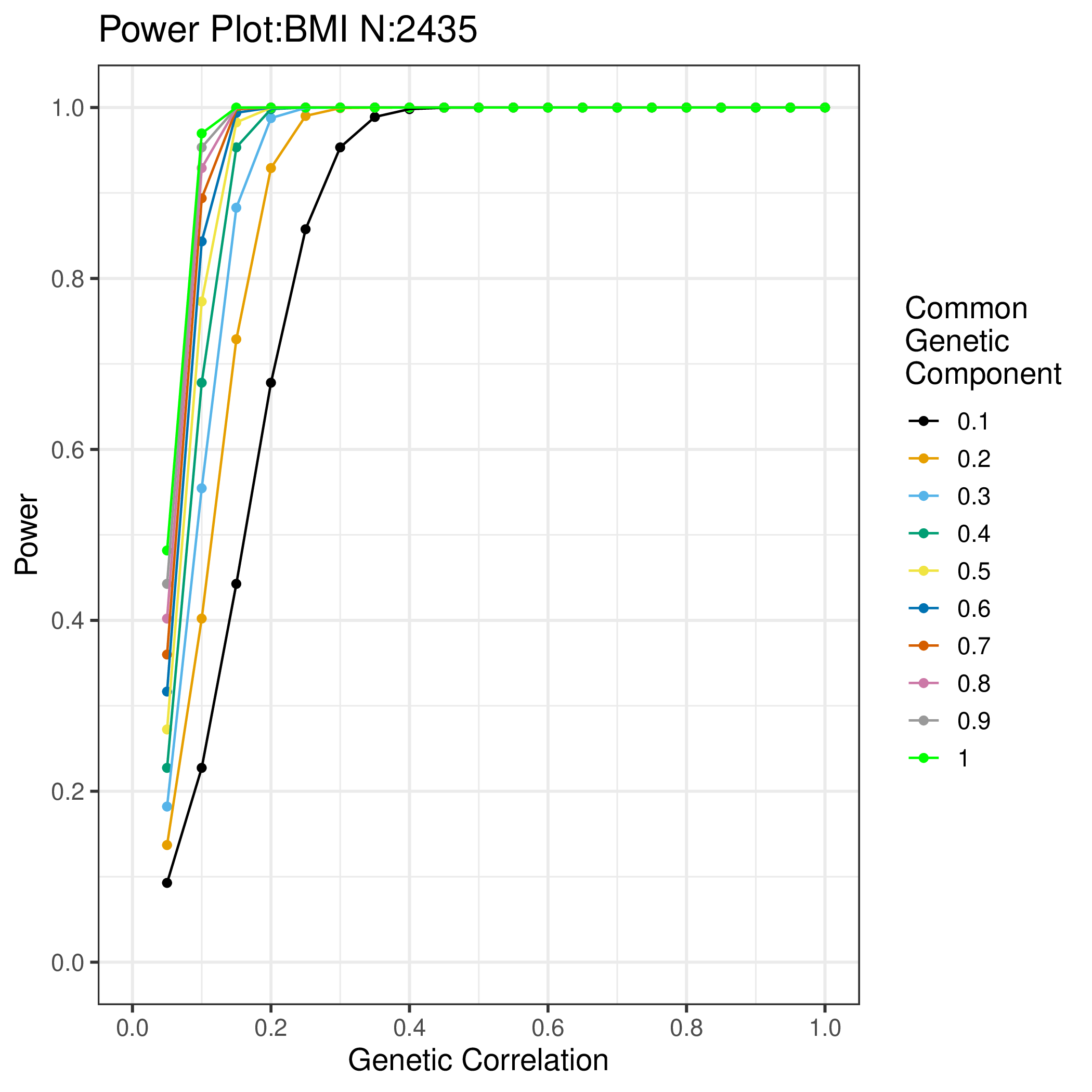
**

Supplementary Figure 4: Power (y axis) to detect a genetic relationship between Height_1_ and a cellular phenotype with a common genetic component of varying size (coloured lines) at different values of genetic correlation (x-axis), for differing values of N:

a) 60 [not accounting for multiple measurements],

b) 850 [lower estimate of effective N],

c) 2435 [higher estimate of effective N].

Supplementary References

1000 Genomes Project Consortium, Auton A, Brooks LD, et al (2015) A global reference for human genetic variation. Nature 526:68–74. doi: 10.1038/nature15393

Chang CC, Chow CC, Tellier LC, et al (2015) Second-generation PLINK: rising to the challenge of larger and richer datasets. Gigascience 4:7. doi: 10.1186/s13742-015-0047-8

Coleman JRI, Euesden J, Patel H, et al (2016) Quality control, imputation and analysis of genome-wide genotyping data from the Illumina HumanCoreExome microarray. Brief Funct Genomics 15:298–304. doi: 10.1093/bfgp/elv037

Core Team R, Others (2013) R: A language and environment for statistical computing. Vienna, Austria: R Foundation for Statistical Computing. Available

Dudbridge F (2013) Power and predictive accuracy of polygenic risk scores. PLoS Genet 9:e1003348. doi: 10.1371/journal.pgen.1003348

Durbin R (2014) Efficient haplotype matching and storage using the positional Burrows-Wheeler transform (PBWT). Bioinformatics 30:1266–1272. doi: 10.1093/bioinformatics/btu014

Faes C, Molenberghs G, Aerts M, et al (2009) The Effective Sample Size and an Alternative Small-Sample Degrees-of-Freedom Method. Am Stat 63:389–399.

Kilpinen H, Goncalves A, Leha A, et al (2017) Common genetic variation drives molecular heterogeneity in human iPSCs. Nature 546:370–375. doi: 10.1038/nature22403

Lee SH, Goddard ME, Wray NR, Visscher PM (2012) A better coefficient of determination for genetic profile analysis. Genet Epidemiol 36:214–224. doi: 10.1002/gepi.21614

Leha A, Moens N, Meleckyte R, et al (2016) A high-content platform to characterise human induced pluripotent stem cell lines. Methods 96:85–96. doi: 10.1016/j.ymeth.2015.11.012

Loh P-R, Danecek P, Palamara PF, et al (2016) Reference-based phasing using the Haplotype Reference Consortium panel. Nat Genet 48:1443–1448. doi: 10.1038/ng.3679

McCarthy S, Das S, Kretzschmar W, et al (2016) A reference panel of 64,976 haplotypes for genotype imputation. Nat Genet 48:1279–1283. doi: 10.1038/ng.3643

Nagelkerke NJD (1991) A note on a general definition of the coefficient of determination. Biometrika 78:691–692. doi: 10.1093/biomet/78.3.691

Palla L, Dudbridge F (2015) A Fast Method that Uses Polygenic Scores to Estimate the Variance Explained by Genome-wide Marker Panels and the Proportion of Variants Affecting a Trait. Am J Hum Genet 97:250–259. doi: 10.1016/j.ajhg.2015.06.005

Vigilante A, Laddach A, Moens N, et al (2019) Identifying Extrinsic versus Intrinsic Drivers of Variation in Cell Behavior in Human iPSC Lines from Healthy Donors. Cell Rep 26:2078–2087.e3. doi: 10.1016/j.celrep.2019.01.094
